# A *Cyclin E* Centered Genetic Network Contributes to Alcohol-Induced Variation in Drosophila Development

**DOI:** 10.1534/g3.118.200260

**Published:** 2018-06-06

**Authors:** Tatiana V. Morozova, Yasmeen Hussain, Lenovia J. McCoy, Eugenea V. Zhirnov, Morgan R. Davis, Victoria A. Pray, Rachel A. Lyman, Laura H. Duncan, Anna McMillen, Aiden Jones, Trudy F. C. Mackay, Robert R. H. Anholt

**Affiliations:** * W. M. Keck Center for Behavioral Biology, Program in Genetics, and Department of Biological Sciences, North Carolina State University, Raleigh, NC 27695-7614; † Department of Biochemistry and Physiology, School of Bioscience and Medicine, Faculty of Health and Medical Sciences, University of Surrey, Guildford, Surrey GU2 7XH, UK; ‡ UNC/NCSU Joint Department of Biomedical Engineering, North Carolina State University, Raleigh, NC 27695-7115

**Keywords:** genome-wide association, genetic networks, ethanol sensitivity, Drosophila Genetic Reference Panel, mutational analysis

## Abstract

Prenatal exposure to ethanol causes a wide range of adverse physiological, behavioral and cognitive consequences. However, identifying allelic variants and genetic networks associated with variation in susceptibility to prenatal alcohol exposure is challenging in human populations, since time and frequency of exposure and effective dose cannot be determined quantitatively and phenotypic manifestations are diverse. Here, we harnessed the power of natural variation in the *Drosophila melanogaster* Genetic Reference Panel (DGRP) to identify genes and genetic networks associated with variation in sensitivity to developmental alcohol exposure. We measured development time from egg to adult and viability of 201 DGRP lines reared on regular or ethanol- supplemented medium and identified polymorphisms associated with variation in susceptibility to developmental ethanol exposure. We also documented genotype-dependent variation in sensorimotor behavior after developmental exposure to ethanol using the startle response assay in a subset of 39 DGRP lines. Genes associated with development, including development of the nervous system, featured prominently among genes that harbored variants associated with differential sensitivity to developmental ethanol exposure. Many of them have human orthologs and mutational analyses and RNAi targeting functionally validated a high percentage of candidate genes. Analysis of genetic interaction networks identified C*yclin E* (C*ycE*) as a central, highly interconnected hub gene. *Cyclin E* encodes a protein kinase associated with cell cycle regulation and is prominently expressed in ovaries. Thus, exposure to ethanol during development of *Drosophila melanogaster* might serve as a genetic model for translational studies on fetal alcohol spectrum disorder.

In humans, prenatal exposure to ethanol causes a wide range of adverse physiological, behavioral and cognitive consequences, including growth deficiency, developmental delay, reduced brain size, and fetal death. These conditions are known as fetal alcohol effects (FAE) or fetal alcohol spectrum disorder (FASD) (Hoyme *et al.* 2005; Manning and Eugene Hoyme 2007; Memo *et al.* 2013).

Rodent models have been used to examine morphological and neurological changes that occur following alcohol exposure, but the mechanisms of those effects are still unclear (Kleiber *et al.* 2011; Kleiber *et al.* 2012; Schambra *et al.* 2015; Marquardt and Brigman 2016; Saito *et al.* 2016). Damage to the heart, brain and skeleton in response to prenatal alcohol exposure has been documented in animal models (Cavieres and Smith 2000; Debelak and Smith 2000; Su *et al.* 2001; Smith *et al.* 2014; Sarmah and Marrs 2017). Studies on chicken embryos and cell lines revealed altered expression of genes related to ribosome biogenesis, mRNA splicing and protein processing, as well as energy metabolism and oxidative phosphorylation (Downing *et al.* 2012; Garic *et al.* 2014; Rogic *et al.* 2016). The nervous system is especially susceptible to developmental alcohol exposure, with widespread transcript abundance changes among genes associated with cell adhesion, synaptogenesis and synaptic signaling (Tyler and Allan 2014; Halder *et al.* 2015; Mandal *et al.* 2015). However, comprehensive population level studies that can accurately assess genotype by exposure effects are impractical for studies in vertebrate animal models.

Identifying allelic variants and genetic networks associated with variation in susceptibility to prenatal alcohol exposure is especially challenging in human populations, due to incomplete or unreliable maternal drinking histories, and the diversity of phenotypic manifestations, some of which may appear after a time lag. *Drosophila melanogaster* has been proposed as a model for FASD (McClure *et al.* 2011; Logan-Garbisch *et al.* 2014), since developmental ethanol exposure leads to reduced viability and developmental delay. Altered expression of insulin-like peptides and their receptors in the brain (McClure *et al.* 2011) as well as oxidative stress (Logan-Garbisch *et al.* 2014) have been implicated as possible mechanisms. Previous studies on the effects of developmental ethanol exposure were, however limited to a few genotypes, focused on selected pathways, and did not provide insights in the genetic underpinnings that determine individual variation in sensitivity to developmental ethanol exposure.

We took advantage of the *Drosophila melanogaster* Genetic Reference Panel (DGRP; Mackay *et al.* 2012; Huang *et al.* 2014) to perform a genome wide association (GWA) analysis to infer candidate genes associated with variation in development time and viability upon ethanol exposure. The DGRP represents a population of fully sequenced, wild-derived, inbred lines with well-annotated genomes. We found extensive variation in viability and development time among DGRP lines grown on regular and on ethanol-supplemented food, with flies developing on average slower when exposed to ethanol. They also showed reduced viability and impaired sensorimotor integration as measured through locomotor reactivity. Analysis of candidate genes revealed a genetic interaction network with *Cyclin E* (*CycE*) as a central hub gene. *CycE* encodes a serine-threonine protein kinase which plays a regulatory role in development and is highly expressed in ovaries (Richardson *et al.* 1993; Richardson *et al.* 1995; Sauer *et al.* 1995). Mutational analyses and RNAi interference experiments provide causal validation for *CycE* and associated genes as developmental targets for ethanol exposure.

## Materials and Methods

### Drosophila stocks

We used 201 DGRP lines (Mackay *et al.* 2012; Huang *et al.* 2014) reared on cornmeal-molasses-yeast medium (hereafter referred to as standard or regular medium) at 25° and 70% humidity under a 12 hr light-dark cycle (lights on at 6:00 am) to measure viability and development time, and a subset of 39 DGRP lines (Ayroles *et al.* 2009) to measure locomotor reactivity. The DGRP consists of 205 lines derived from a natural population from North Carolina by 20 generations of full-sib inbreeding followed by whole genome sequencing to high coverage (Mackay *et al.* 2012). For functional validation seven *P{MiET1}* mutants and their co-isogenic control *w^1118^_iso_*; *2_iso_*; *3_iso_* (Bellen *et al.* 2011) were obtained from the Bloomington *Drosophila* stock center (*bab1*, *CG17150*, *CG42820*, *CG43729*, *CG6024*, *Nek2*, *nuf* and *SKIP*; Bloomington, IN). In addition, we obtained 18 RNAi transgenic fly strains of the *phiC31* (KK) RNAi library (*CG1440*, *CG32264*, *CCG34351*, *CG34370*, *CG43894*, *CycE*, *dve*, *Egfr*, *fd59A*, *ft*, *fz*, *Lim1*, *mam*, *msn*, *pbl*, *sgg*, *ZnT41F* and *zormin*), together with the corresponding progenitor line (60010) from the Vienna Drosophila RNAi Center (VDRC; Dietzl *et al.* 2007). These lines and the appropriate progenitor controls were crossed to a weak *Ubiquitin-GAL4* driver to suppress the expression of the gene of interest in hybrid F1 offspring (Garlapow *et al.* 2015).

### Viability and development time

To measure viability and development time we allowed parents to lay eggs overnight. The next day we collected eggs from the parental vials and placed 50 eggs per replicate per line on standard medium or on medium supplemented with 10% (v/v) ethanol. We used five replicate vials per line and per medium for measurement of development time and viability. To account for ethanol evaporation all the vials were kept in behavioral chambers with controlled humidity and temperature and all DGRP lines were exposed to the same condition. In addition experimental replicates were done with a randomized design to avoid batch effects. Flies were not exposed to CO_2_ anesthesia for at least 24 hr prior to the assay. To measure development time we collected and counted eclosing adult flies every morning between 9:00 and 10:00 am. We used the mean eclosion day across all flies as a measurement of development time for each line. We used the fraction of surviving adults out of 50 eggs as a measurement of viability. Sensitivity to ethanol was determined as the difference in viability or development time between flies grown on ethanol-supplemented and regular food.

### Locomotion

Locomotor reactivity was measured as startle behavior for 39 DGRP lines, as described previously (Yamamoto *et al.* 2008). A single three- to five-day-old fly grown on ethanol or regular food was placed in a vial and subjected to a mechanical disturbance by quickly tapping the vial twice on the bench top. The vial was placed horizontally, and the locomotor score was recorded as the amount of time the fly remained mobile within a 60 s period immediately following the disturbance. This assay was performed with 20 replicate measurements per line per sex and per condition.

### Quantitative genetic analyses

We used mixed model factorial analysis of variance (ANOVA) to partition variance in replicate means of development time and viability to ethanol exposure among the DGRP lines, according to the model *Y* = *μ* + *T* + *L* + *LxT* + ε, where *μ* is the overall mean, *T* is the fixed effect of treatment (ethanol or regular), *L* represents line (random), *LxT* is the interaction term (random) and *ε* is the within line (error) variance. We used a reduced ANOVA of form *Y* = *μ* + *L* + *ε* for each growth condition separately. We estimated variance components from the full model using the restricted maximum likelihood method and calculated broad sense heritability as *H*^2^ = *σ^2^_G_/σ^2^_P_*, where *σ^2^_G_* is the total genetic variation (*σ*^2^*_L_* + *σ*^2^*_LT_)* and *σ^2^_P_* is the total phenotypic variation (*σ*^2^*_L_* + *σ*^2^*_LT_* + *σ*^2^*ε*).

For locomotor behavior we obtained data for both sexes; therefore, we partitioned the variance using the ANOVA model: *Y* = *μ* + *T* + *S* + *L* + *TxS + LxS + LxT + LxTxS* + ε, where *μ* is the overall mean, *T* is the fixed effect of treatment (ethanol or regular), *L* represents line (random), *S* is the fixed effect of sex (females or males), *TxS*, *LxS*, *LxT and LxTxS* are the interaction terms and *ε* is the within line (error) variance. We used a reduced ANOVA of form *Y* = *μ* + *S* + *L* + *LxS* + *ε* for each growth condition separately. We calculated the broad-sense heritability of locomotor reactivity from the full model as *H*^2^ = *σ^2^_G_/σ^2^_P_ =* (*σ*^2^*_L_*+ *σ*^2^*_LT_ + σ*^2^*_LS_* + *σ*^2^*_LTS_*) / (*σ*^2^*_L_*+ *σ*^2^*_LT_ + σ*^2^*_LS_* + *σ*^2^*_LTS_*+ *σ*^2^*ε*).

### Genome-wide association analysis

We performed GWA analyses for development time and viability on each rearing medium, as well as sensitivity, using a mixed linear model implemented using the pipeline available at http://dgrp2.gnets.ncsu.edu. GWA analyses were performed on line means using 1,876,781 variants that are present at minor allele frequencies of at least 0.05. The effects of *Wolbachia pipientis* infection, common polymorphic inversions, and polygenic relatedness, were taken into account, as described previously (Mackay *et al.* 2012, Huang *et al.* 2014).

### Bioinformatics analyses

We annotated DNA variants using the gene models in Flybase release r5.57 (McQuilton *et al.* 2012). We downloaded the complete genetic interaction networks from FlyBase (release r5.57). The genes in the networks are represented as nodes, whereas edges between the nodes represent interactions. We mapped candidate genes significant at *P* < 5x10^−5^ from GWA analyses for viability, development time, and all candidate genes combined from both traits to the graphical interface of genetic networks using the *igraph* package in R (R Core Team 2016). We then extracted subnetworks from the global networks whose edges were either a direct connection between candidate genes or bridged by only one gene not among the candidate gene list. We evaluated the significance of the size of the largest cluster among the subnetworks by a randomization test in which we randomly extracted subnetworks with the same number of input genes. The *P*-value was determined by dividing the number of instances where the size of the largest cluster exceeds the observed largest size by the total number of randomizations (*α*=0.05) (Antonov *et al.* 2008; Carbone *et al.* 2016; Fochler *et al.* 2017).

We performed Gene Ontology enrichment analyses using DAVID software (Huang *et al.* 2009). Human orthologs were obtained using the DRSC Integrative Ortholog Prediction Tool (DIOPT, version 5.4; http://www.flyrnai.org/diopt; Hu *et al.* 2011). A gene interaction network for human orthologs was constructed using R-Spider (http://www.bioprofiling.de; Antonov 2011).

### Functional analyses of candidate genes

#### Viability and development time:

We selected nine (*CG1440*, *CG34370*, *CG42820*, *CG43729*, *CG43894*, *CG6024*, *msn*, *nuf* and *SKIP*) and ten (*bab1*, *CG17150*, *CG32264*, *CG34351*, *dve*, *fd59A*, *Lim1*, *Nek2*, *ZnT41F* and *zormin*) genes for association with alcohol-dependent variation in viability and development time at *P* < 10^−6^, respectively. In addition, we functionally tested the six most connected genes in the network associated with phenotypic variation (*CycE*, *ft*, *fz*, *mam*, *msn* and *sgg*) along with two computationally predicted genes (*Egfr* and *pbl*). Viability and development time were measured for all genotypes as described above for the DGRP lines, but with 10 replicate vials per genotype.

#### Locomotion:

Locomotor behaviors were quantified using two different assays, startle induced locomotor reactivity (Yamamoto *et al.* 2008) and negative geotaxis, for the subset of highly interconnected candidate genes (*CycE*, *ft*, *fz*, and *sgg*). We analyzed negative geotaxis based on the countercurrent apparatus designed by Benzer (Benzer 1967). We collected 50 3-5 day old flies of the same sex per replicate and performed five replicate assays for mutant lines and their control, reared on ethanol or regular medium. Flies were allowed to recover overnight from CO_2_ anesthesia. To begin the assay, flies were tapped to the bottom of the first start tube, and the apparatus was positioned vertically. The flies were given 15 s to reach the distal tube. This procedure was repeated seven times, such that flies could choose to go upward a maximum of eight times. At the end of the trial, all eight start tubes containing flies were removed and frozen at −20° before manually counting the flies in each tube. Each individual fly is assigned a score from 0 (did not move up) to 7 (moved up every trial) (Carbone *et al.* 2016).

#### Alcohol sensitivity:

Alcohol sensitivity was measured for a *CycE* –targeted RNAi knockdown line compared to its control as 50% sedation by collecting 10 replicates of 8 flies (3-7 days old) of the same sex per replicate and adding 1 ml of ethanol solution onto a vial plug. The number of intoxicated flies, which lost postural control, was recorded every 2 min. The observation time ended when at least 4 flies became intoxicated (Maples and Rothenfluh 2011).

#### Data analyses:

We analyzed sensitivity to ethanol exposure by comparing differences in development time, viability, startle-induced locomotor reactivity, negative geotaxis and alcohol sensitivity between ethanol supplemented and regular medium and between mutant lines and appropriate controls, using a mixed model ANOVA of form *Y* = *μ* + *T* + *L* + *T*×*L* + *ε*, where *T* indicates the fixed effect of treatment (ethanol *vs.* regular medium), *L* indicates mutant or control genotypes (fixed) and *ε* is the residual variance. For startle-induced locomotor reactivity, negative geotaxis, and alcohol sensitivity we performed data analyses for sexes separately. Significance of the *T*×*L* interaction term indicates an effect of the mutation on the sensitivity to ethanol exposure for a given trait.

### Data availability

DGRP lines are publicly available from the Bloomington stock center, IN. Raw phenotypic data for line means are presented in Table S1. Supplemental material is available at Figshare: https://doi.org/10.25387/g3.6213629.

## Results

### Variation in development time and viability of dgrp lines upon ethanol exposure

**Figure 1:**
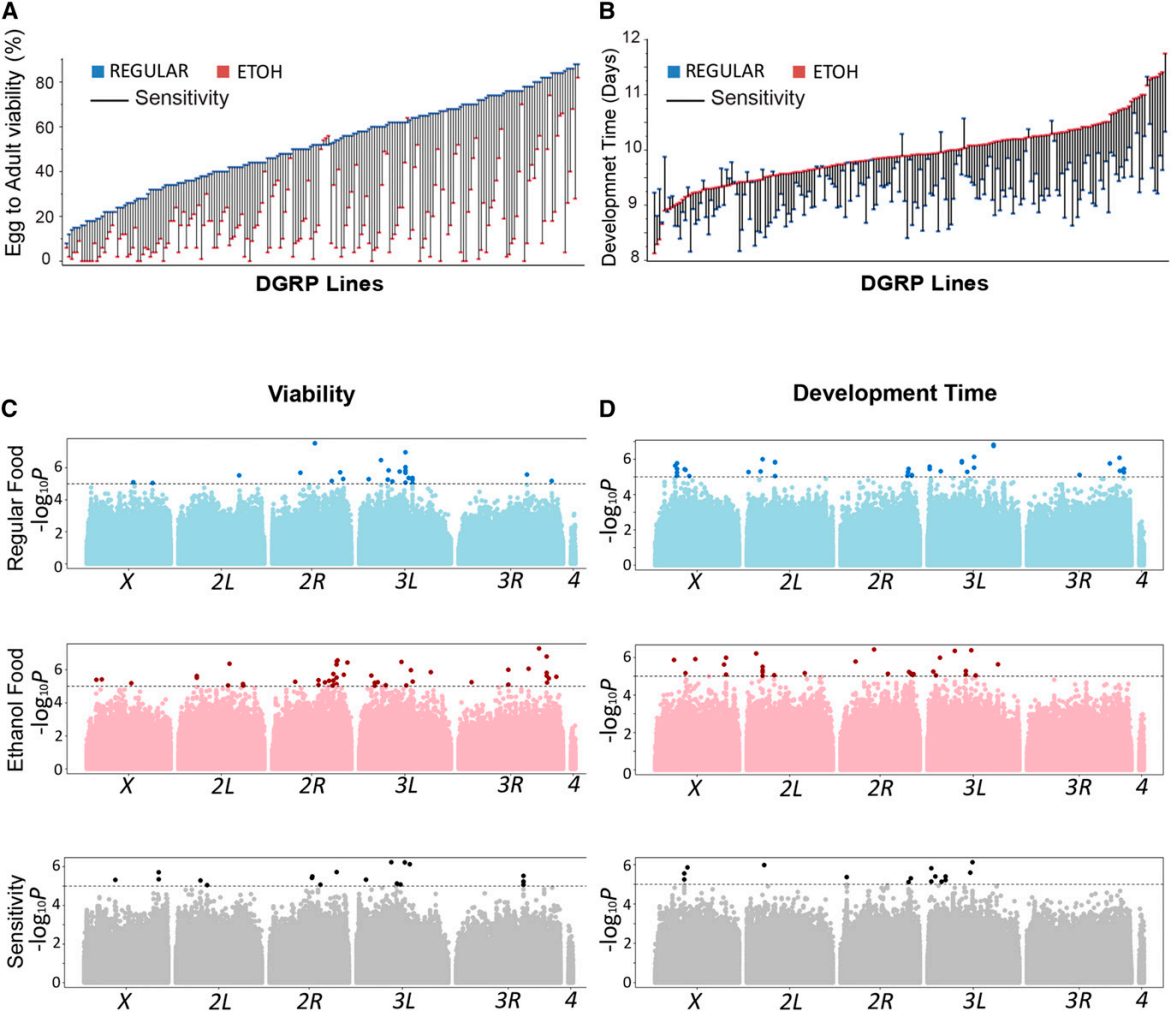
Phenotypic variation and genome-wide associations for viability, development time and sensitivity to ethanol exposure among 201 DGRP lines. (A) Distribution histogram for variation in viability. (B) Distribution histogram for variation in development time. Blue symbols in panels A and B indicate growth on standard medium and red symbols indicate growth on medium supplemented with 10% ethanol. The differences between the two growth conditions, illustrated by the black connecting lines, represent the sensitivity to ethanol exposure. (C) GWA analysis for viability for flies reared on regular food (top panel), ethanol-supplemented medium (middle panel) and the difference, reflecting sensitivity (lower panel). (D) GWA analysis for development time for flies reared on regular food (top panel), ethanol-supplemented medium (middle panel) and the difference, reflecting sensitivity (lower panel). Sensitivity to ethanol was determined as the difference in viability or development time between flies grown on ethanol-supplemented and regular food. The *X*-axes in (C) and (D) indicate chromosomal locations. The dashed lines correspond to the *P* < 10^−5^ statistical threshold. Darker dots above the line indicate SNPs that pass the statistical threshold.

We measured development time and egg-to-adult survival of 201 DGRP lines reared on standard medium and medium supplemented with 10% ethanol. Viability was measured as the fraction of eggs that hatched and developed to adults. We observed extensive variation in viability across the lines, with overall reduced viability when flies were reared on ethanol (Figure 1A). Analyses of variance show significant Line by Treatment interactions, indicating that sensitivity to ethanol exposure as measured by viability or development time is dependent on genetic background (Tables S2 and S3). Broad sense heritability estimates were 0.54 for growth conditions combined and 0.46 for growth on ethanol-supplemented as well as regular medium, showing a substantial genetic contribution to the observed variation (Table S2). However, differences between viability on regular food and ethanol-supplemented food varied greatly for each individual line, indicating substantial variation in sensitivity to developmental alcohol exposure (Figure 1A). The mean viability for flies was 52% reared on standard medium and 20% on ethanol supplemented medium. Sensitivity to ethanol, estimated as the difference of survival between growth on ethanol-supplemented food *vs.* standard food, ranged from 0 to 100%, with a mean of 37%.

Development time was measured as the number of days it takes for an egg to develop to adult for 198 of the 201 lines that survived on both ethanol-supplemented and standard medium. Mean eclosion time was 9.3 days for flies reared on standard food and 9.9 days (one half day later) for flies reared on ethanol (Figure 1B). Surprisingly, while on average flies grown on ethanol developed more slowly than those reared on standard medium, some lines developed faster on ethanol-supplemented food (Figure 1B). Heritability estimates were ∼0.3 for growth on ethanol-supplemented as well as regular medium (Table S3). Again, there was wide variation in sensitivity to ethanol exposure, *i.e.*, the difference between development time on ethanol-supplemented food and regular medium. Whereas some lines appeared marginally or not at all affected by this concentration of ethanol, three of the lines were not viable. These lines were excluded from subsequent analyses. Sensitivity of development time, calculated as the difference in mean development time on ethanol-supplemented *vs.* standard food, ranged from -1.1 to 2.1 days, with an average sensitivity of 0.6 days (Figure 1B).

The correlation between sensitivity to ethanol exposure for viability and development time was not statistically significant (*r* = 0.08, *P* = 0.25). However, we found significant positive correlations between development time (*r* = 0.47; *P* < 0.0001) and viability (*r* = 0.48; *P* < 0.0001) on ethanol-supplemented *vs.* regular media (Figure S1).

### Phenotypic variation in startle-induced locomotor activity after developmental ethanol exposure

**Figure 2:**
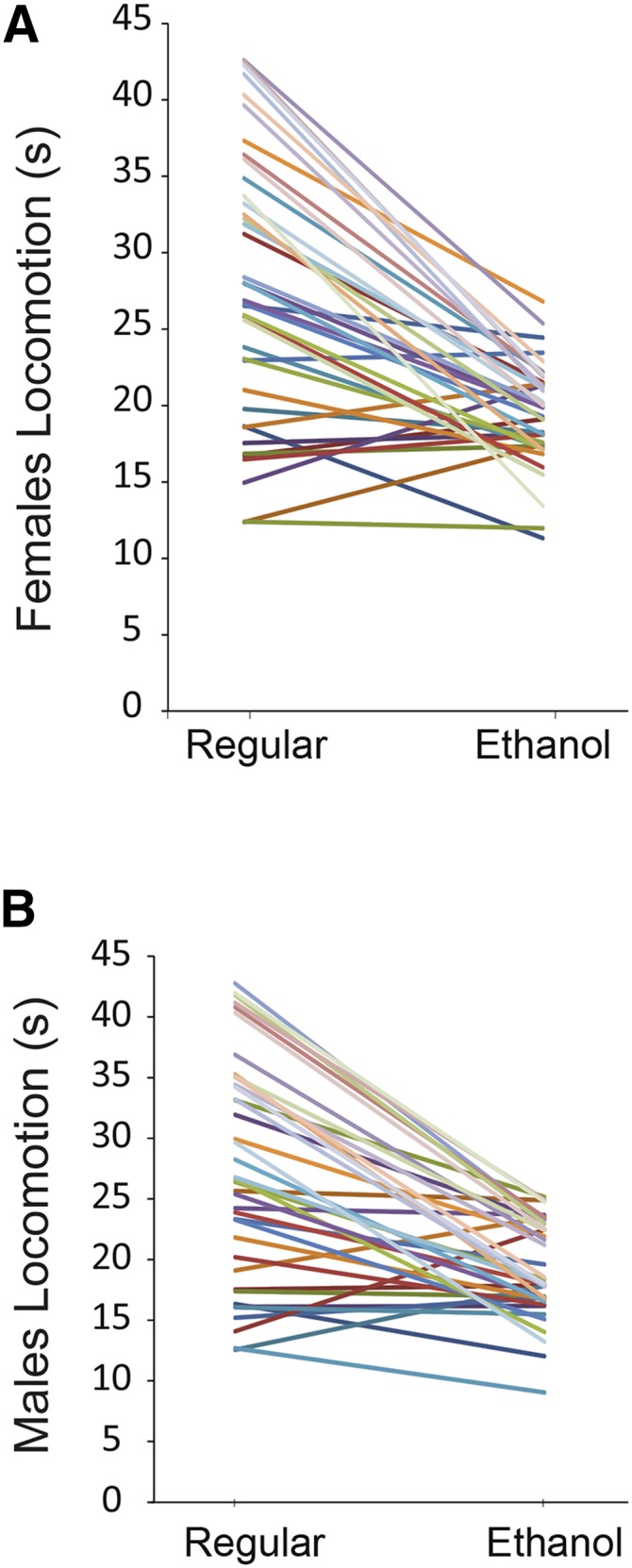
Reaction norms that illustrate variation in startle-induced locomotor activity among 39 DGRP lines grown on regular and ethanol supplemented media for females (A) and males (B). Colors represent the different DGRP lines.

To assess the effect of developmental alcohol exposure on sensorimotor behavior, we measured startle-induced locomotor activity in a subset of 39 DGRP lines. On average, startle-induced locomotor activity was higher when flies were reared on regular food and reduced when reared on ethanol-supplemented medium, indicating compromised sensorimotor function (Figure 2). We did not observe significant differences between males and females on average, although there was significant genetic variation in sexual dimorphism of locomotor behavior (Table S4). The heritability estimate for the two growth conditions combined was 0.64 (Table S4).

### Gwa analyses for development time and viability

We performed single marker genotype-phenotype association analyses using line means for viability and development time on regular and ethanol-supplemented food, and for sensitivity for these traits using the DGRP web portal (http://dgrp2.gnets.ncsu.edu). We tested 1,891,456 variants with minor allele frequencies greater than 0.05, after accounting for effects of *Wolbachia* infection, common polymorphic inversions, and polygenic relatedness, and identified alleles associated with alcohol sensitivity for development time and viability at a nominal threshold of *P* < 10^−5^. Quantile-quantile plots showed deviations of observed from expected values at this significance level (Figure 1, C and D, Figure S2).

We identified 26 polymorphisms in 19 genes associated with viability on regular food, 47 variants located in or near 33 genes that were associated with phenotypic variation in viability on ethanol supplemented food, and 19 polymorphisms in or near 12 genes that contribute to sensitivity of viability (Figure 1C, Table S5). There was no overlap between these SNPs. Candidate genes associated with variation in viability for all conditions represent Gene Ontology categories associated with cell morphogenesis, axonogenesis, and neuron development. At a relaxed *P* value < 5 x10^−5^, the number of associated SNPs increased to 284, located in or near 194 genes, and with more input genes Gene Ontology enrichment analysis revealed neuron development, nervous system development, chemotaxis, and organ morphogenesis as significant categories (Table S6).

We identified 37 variants in 27 genes associated with variation for development time when flies were reared on regular food, 30 polymorphisms located in or near 23 genes that were associated with variation for development time on ethanol supplemented food, and 15 polymorphisms in or near 14 genes that contributed to variation in sensitivity of development time (Figure 1D, Table S7). There was no overlap between these SNPs. At *P* < 5x10^−5^ we identified 198 SNPs located in or near 138 genes. Gene Ontology enrichment analysis revealed *Wnt* signaling, signal transduction, tissue morphogenesis and neuron development, associated with variation in developmental time on ethanol-supplemented food (Table S8).

### Functional analysis of candidate genes from the gwa analyses

**Figure 3:**
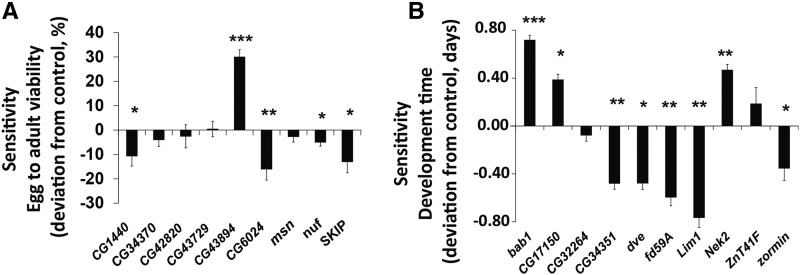
Functional validation of candidate genes associated with variation in sensitivity to ethanol exposure for viability (A) and development time (B). Data are shown as differences between viability or development time on ethanol-supplemented food *vs.* regular food and presented as deviation from the appropriate control ± SE * *P* < 0.05; ** *P* < 0.001; *** *P* < 0.0001.

We conducted functional analyses of candidate genes using either RNAi knockdown of gene expression or *P{MiET1}* insertional mutants corresponding to candidate genes that harbor SNPs associated with sensitivity to ethanol exposure for development time or viability at *P* < 10^−6^ (Tables S5 and S7). To separate the effects of developmental mutations from mutations affecting sensitivity to ethanol exposure, we measured viability and development time on regular and ethanol-supplemented media for the mutants and their corresponding controls. Among the nine mutants tested, five (56%) showed a significant difference in viability from the control (Figure 3A; Table S9A). Mutations in *CG1440*, *CG6024*, *nuf* and *SKIP* caused a decrease in viability, while the *CG43894* mutant significantly increased viability when grown on ethanol. This indicates that the wild type *CG43894* allele limits viability on exposure to ethanol. *CG1440* is predicted to be involved in proteolysis and response to toxic substance (Mi *et al.* 2010); *nuf* is associated with actin cytoskeleton reorganization (Riggs *et al.* 2003; Cao *et al.* 2008); *CG6024* and *CG43894* have unknown functions. *SKIP* has been implicated in sensory perception of smell (Tunstall *et al.* 2012).

We tested ten mutants/RNAi lines for effects of ethanol exposure on development time, and found that eight of them showed a significant difference between growth on ethanol and regular medium (Figure 3B; Table S9B). *bab1*, *CG17150* and *Nek2* mutants showed an increase in development time, while *CG34351*, *dve*, *fd59A*, *Lim1* and *zormin* developed faster on ethanol. These genes are involved in a wide range of biological processes. *bab1* is associated with cuticle pigmentation (Kopp *et al.* 2000; Dembeck *et al.* 2015); *dve* is involved in midgut (Nakagawa *et al.* 2011) and reproductive structure development (Minami *et al.* 2012); *fd59A* plays role in controlling egg-laying behavior (Lacin *et al.* 2014); *Lim1* is associated with eye development and regulation of transcription and gene expression (Kojima *et al.* 2005; Roignant *et al.* 2010); *CG17150* plays a role in sperm competition (Karak *et al.* 2015); *Nek2* contributes to regulation of mitotic nuclear division (Prigent *et al.* 2005) and protein phosphorylation (Schertel *et al.* 2013); *CG34351* and *zormin* have unknown functions.

### A Genetic interaction network for sensitivity to developmental alcohol exposure

We asked to what extent the 254 genes (Tables S5 and S7) associated with variation in viability on regular or ethanol-supplemented media as well as sensitivity to ethanol at *P* < 5x10^−5^ participate in known gene-gene interactions. We identified a network comprised of 42 interacting candidate genes and 82 computationally recruited genes (Figure S3A). Gene ontology analysis of 124 interacting genes showed significant enrichment of organ and tissue morphogenesis, and cell development, including nervous system and sensory organ development (Table S10). *CycE*, *fz*, *mam*, *msn* and *sgg* were the most connected candidate genes.

Similarly, we assessed to what extent the 224 (Tables S3 and S5) genes associated with variation in development time on regular or ethanol-supplemented media as well as sensitivity to ethanol (*i.e.*, the difference in development time between the two growth conditions) at *P* < 5x10^5^, participate in known gene-gene interactions. Here, we identified a network of 39 interacting candidate and 44 computationally recruited genes (Figure S3). Gene ontology analysis of 83 interacting genes showed significant enrichment of genes associated with cell development and nervous system development, organ and tissue morphogenesis, and regulation of signal transduction (Table S11). *Bx*, *ft*, *kuz* and *sgg* were the most highly connected candidate genes.

**Figure 4:**
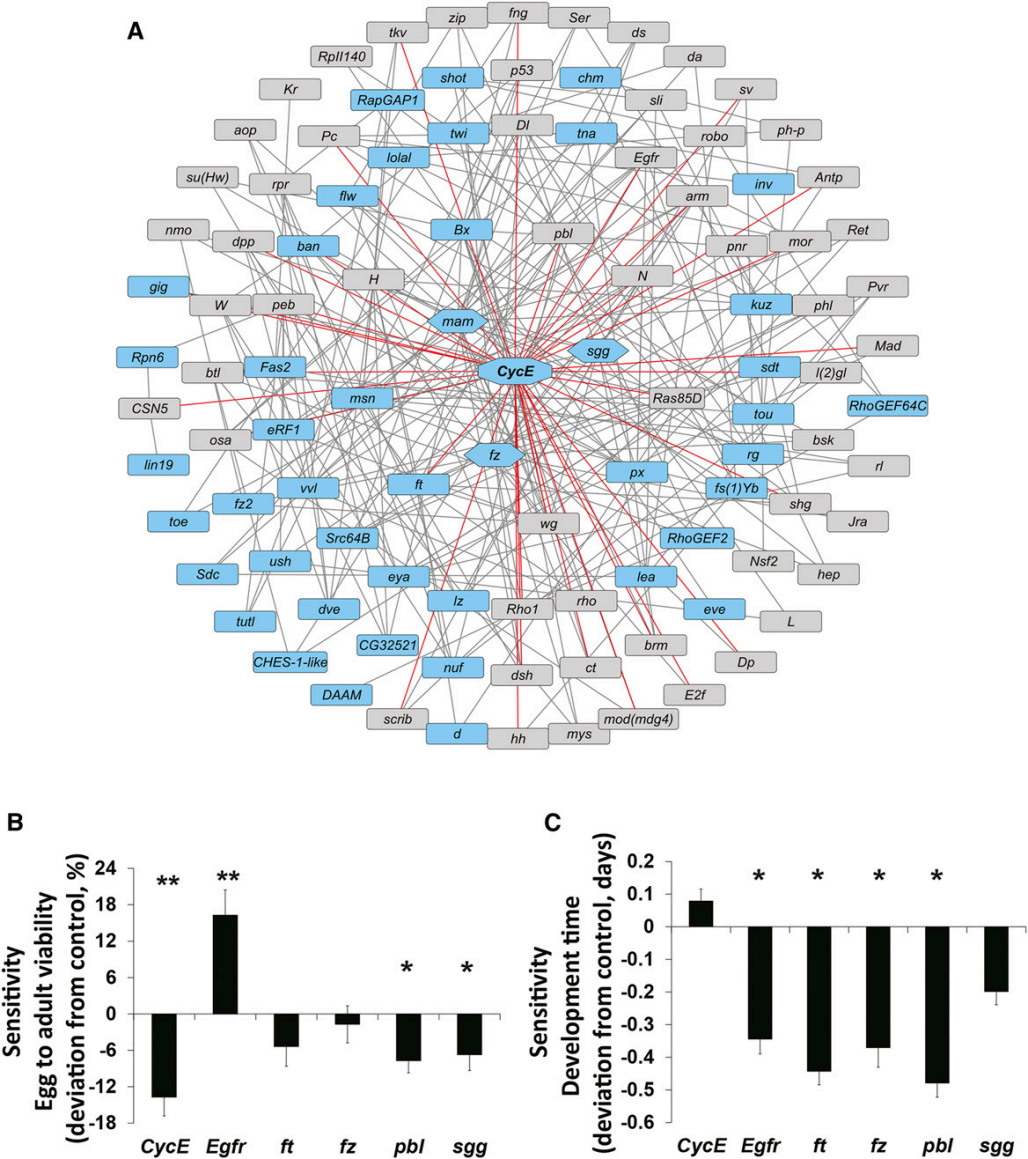
A genetic interaction network for ethanol-dependent variation in viability and development time. (A) The network consists of candidate genes identified by the GWA analysis at *P* < 5 × 10^−5^. We identified 184 interconnected genes (*P* < 0.001) with 78 candidate genes and 106 computationally recruited genes, *i.e.*, genes that were not identified in our study, but are known to interact with our candidate genes based on published data curated in FlyBase. The network shows only genes with three or more known genetic interactions. The octagon shape in the center indicates the most highly connected *CycE* gene with red lines highlighting its 54 nodal connections. Hexagons indicate the additional highly connected genes *mam*, *sgg* and *fz*. Blue rectangular boxes indicate candidate genes identified by GWA analyses and gray boxes represent computationally recruited genes. (B) Functional validation of highly connected candidate genes from the network analysis for viability. (C). Functional validation of highly connected candidate genes from the network analysis for development time. * *P* < 0.05 and ** *P* < 0.001 indicates a significant difference between sensitivity in RNAi knockdown lines grown on ethanol-supplemented food *vs.* regular food. Data are shown as deviation from the appropriate control ± SE.

The genetic interaction networks for viability and development time (Figures S7 and S8) did not reach statistical significance individually. However, since viability and development time are related traits, we combined the candidate genes from both GWA analyses and constructed a network using 461 input genes with a significance threshold of *P* < 5x10^−5^ (Figure 4). This network was significant compared to the probability of obtaining the same size network by chance (*P* < 0.001) and consisted of 184 interconnected genes with 78 candidate genes and 106 computationally recruited genes. For clarity, the network depicted in Figure 4 shows only genes with three or more known genetic interactions.

*CycE*, *fz*, *ft*, *mam* and *msn* were the most connected candidate genes. *CycE* stands out with 49 genetic interactions. *CycE* is associated with variation in viability of flies grown on ethanol-supplemented food, and in the network for development it appears as a computationally recruited gene (Figure 4A). This gene has previously been identified in a network for variation in alcohol sensitivity of adult flies (Morozova *et al.* 2015). Gene ontology analysis of 184 interacting genes from this combined analysis revealed similar enrichment of developmental genes, including development of sensory organs and oogenesis (Table S12).

### Functional analysis of hub genes from the network

Next we assessed the effects on alcohol sensitivity of RNAi knockdown of hub genes in the combined network analysis (Figure 4, B and C; Table S9C and S9D). We measured both viability and development time for eight mutants of highly connected genes in the network (*CycE*, *Egfr*, *ft*, *fz*, *mam*, *msn*, *pbl* and *sgg*). We assessed the effect of RNAi knockdown for the hub genes *CycE*, *sgg*, *mam* and *fz* using a weak *Ubiquitin-GAL4* driver by qRT-PCR and found significant reduction (10–30%) in expression levels in the RNAi knockdown background compared to the control. Reduction in *msn* expression has been confirmed previously (Fochler *et al.* 2017).

We determined sensitivity to ethanol as the differences in viability or development time between flies grown on ethanol-supplemented food and regular food (Figure 4). We confirmed functional associations for sensitivity to ethanol for viability for *CycE*, *Egfr*, *pbl* and *sgg*, with *Egfr* mutants showing increased viability when flies were grown on ethanol containing food compared to regular medium. In contrast, sensitivity to ethanol for development time increased for *Egfr*, *ft*, *fz* and *pbl* mutants, which developed more slowly on ethanol food compared to regular medium. We did not observe significant differences for either trait for *mam* and *msn* mutants.

**Figure 5:**
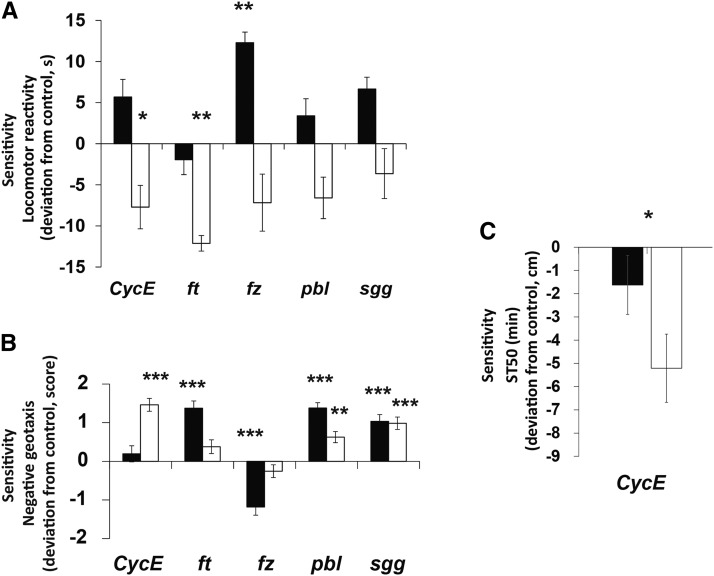
Pleiotropic effects of the hub candidate genes. (A) Locomotor reactivity. (B) Locomotion. (C) Alcohol sensitivity. Black bars indicate females, white bars indicate males. Data are shown as deviation from the appropriate control ± SE * *P* < 0.05; ** *P* < 0.001; *** *P* < 0.0001.

Finally, we used the same RNAi lines to evaluate pleiotropic effects of the hub candidate genes on locomotion and adult ethanol sensitivity. Adult flies exhibit altered behavioral responses when grown on ethanol-supplemented medium, including changes in locomotor behavior and sensitivity to ethanol vapors. We used two different assays to measure locomotor behaviors, startle-induced locomotor reactivity and negative geotaxis (Figure 5). Startle-induced locomotion showed antagonistic sexual dimorphism with tendencies to decrease in males and increase in females. However, in female mutants an increase in the startle response was only significant for *fz*, whereas in male mutants statistically significant declines in startle behavior were observed for *CycE* and *ft* (Figure 5A). In contrast to startle behavior, negative geotaxis was strongly affected in at least one sex of all mutant lines (Figure 5B). With the exception of *fz*, the mutants became more active when grown on ethanol. Knock-down time as a measure of alcohol sensitivity was altered only in the *CycE* mutant males (Figure 5C).

**Figure 6:**
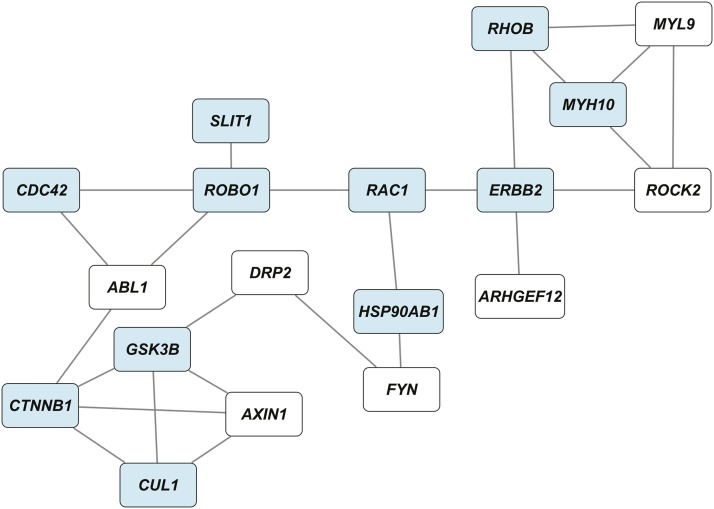
A genetic interaction network of human orthologs corresponding to Drosophila candidate genes. Human orthologs were identified as counterparts to Drosophila genes depicted in Figure 4A. Blue rectangles indicate genes previously implicated in alcohol-related phenotypes in model systems and humans. The probability to obtain this network by chance is *P* < 0.005.

Among candidate genes from the network depicted in Figure 4A, 92% have human orthologs and 18 of them formed a network of interconnected candidate genes (*P* < 0.005; Figure 6). This network is enriched for genes associated with signal transduction, regulation of protein metabolic processes, and central nervous system development, including brain development (Table S13).

## Discussion

We harnessed the power of natural variation captured by the *Drosophila melanogaster* Genetic Reference Panel to identify genes and genetic networks associated with sensitivity to developmental alcohol exposure. Despite the limited size of the DGRP, we were able to identify allelic variants at a lenient *P*-value. We could then use mutational analyses using transposon-tagged mutants and RNAi to assess whether genes that harbor these allelic variants affect the phenotype.

A previous study also reported that flies grown on ethanol show delayed development, decreased viability, reduced adult size, increased locomotion, and resistance to ethanol vapors (McClure *et al.* 2011). Developmental delay and reduced viability in this study were attributed to ethanol-mediated inhibition of the insulin signaling pathway. Whereas we were able to document phenotypic variation in locomotor behaviors that depend on sensorimotor integration among 39 DGRP lines, this sample did not provide enough power for GWA analysis. Our study also did not identify variants in genes of the insulin signaling pathway associated with variation in ethanol sensitivity in the DGRP, but instead identified a wide range of developmental genes, in line with previous cell-based or animal model studies (Mandal *et al.* 2015; Schambra *et al.* 2015; Fish *et al.* 2017).

Examination of the genetic interaction network associated with variation in developmental alcohol sensitivity identifies *Cyclin E* as a central hub gene. The gene product of this highly interconnected gene is a protein kinase associated with cell cycle regulation and oogenesis (Richardson *et al.* 1993; Richardson *et al.* 1995; Sauer *et al.* 1995). *CycE* regulation is crucial for proper S to G phase transition (Shcherbata *et al.* 2004). Expression of *CycE* is controlled by Myc, a transcriptional regulator implicated in the biosynthesis of ribosomes and important for growth and proliferation during normal development (Gallant 2009; Bellosta and Gallant 2010; Quinn *et al.* 2013). Thus, altered *CycE* transcript abundance could result in a wide range of phenotypic effects through an indirect influence on ribosome biosynthesis and, hence, protein synthesis. It is of interest to note that high levels of *CycE* expression occur in ovaries, supporting the notion that developmental exposure to ethanol in Drosophila could serve as a model for fetal alcohol spectrum disorder.

Studies on rodents have proposed that prenatal alcohol exposure can give rise to epigenetic modifications (Perkins *et al.* 2013; Brown and Feng 2017; Chater-Diehl *et al.* 2017; Tulisiak *et al.* 2017). Future studies in Drosophila can assess whether prenatal alcohol exposure results in histone modifications that drive altered gene expression and, if so, to what extent such modifications persist across generations.

Human orthologs can be superimposed on genes contained within the Drosophila genetic interaction network (Figure 6). More than half of these have been previously implicated with effects of developmental ethanol exposure in cell culture models (*ERBB2*, *RAC1*, *ROBO1*, *SLIT1* (Tyler and Allan 2014); *CDC42*, *CTNNB1*, *HSP90AB1*, *ROBO1* (Garic *et al.* 2014));, in chicken embryos (*CTNNB1*, *CDC42*, *GSK3B*, *HSP90AB1*, *MYH10* (Berres *et al.* 2017)); in human embryonic cells (*RHOB* (Mandal *et al.*, 2015)); in a GWA study for alcohol dependence in people (*CTNNB1*, *SLIT1* (Edenberg *et al.* 2010)); and in gene expression microarray analysis of human frontal cortex (*HSP90AB1* (Lewohl *et al.* 2000); *CUL1* (Liu *et al.* 2016)).

In conclusion, exploration of the genetic underpinnings of developmental sensitivity to alcohol exposure in Drosophila has pinpointed *CycE* as a central hub gene and identified human orthologs of Drosophila candidate genes for future studies on human populations to uncover risk alleles for FASD.

## References

[bib1] Antonov A. V. Schmidt T. Wang Y. Mewes H. W., 2008 ProfCom: a web tool for profiling the complex functionality of gene groups identified from high-throughput data. Nucleic Acids Res. 36: W347–W351. 10.1093/nar/gkn239 18460543 PMC2447768

[bib3] Antonov A. V., 2011 BioProfiling.de: analytical web portal for high-throughput cell biology. Nucleic Acids Res. 39: W323–W327. 10.1093/nar/gkr372 21609949 PMC3125774

[bib4] Ayroles J. F. Carbone M. A. Stone E. A. Jordan K. W. Lyman R. F., 2009 Systems genetics of complex traits in *Drosophila melanogaster*. Nat. Genet. 41: 299–307. 10.1038/ng.332 19234471 PMC2752214

[bib5] Bellen H. J. Levis R. W. He Y. Carlson J. W. Evans-Holm M., 2011 The Drosophila gene disruption project: progress using transposons with distinctive site specificities. Genetics 188: 731–743. 10.1534/genetics.111.126995 21515576 PMC3176542

[bib6] Bellosta P. Gallant P., 2010 Myc function in Drosophila. Genes Cancer 1: 542–546. 10.1177/1947601910377490 21072325 PMC2976539

[bib7] Benzer S., 1967 Behavioral mutants of Drosophila isolated by countercurrent distribution. Proc. Natl. Acad. Sci. USA 58: 1112–1119. 10.1073/pnas.58.3.1112 16578662 PMC335755

[bib8] Berres M. E. Garic A. Flentke G. Smith S. M., 2017 Transcriptome profiling identifies ribosome biogenesis as a target of alcohol teratogenicity and vulnerability during early embryogenesis. PLoS One 12: e0169351 10.1371/journal.pone.0169351 28046103 PMC5207668

[bib9] Brown A. N. Feng J., 2017 Drug addiction and DNA modifications. Adv. Exp. Med. Biol. 978: 105–125. 10.1007/978-3-319-53889-1_6 28523543

[bib10] Cao J. Albertson R. Riggs B. Field C. M. Sullivan W., 2008 Nuf, a Rab11 effector, maintains cytokinetic furrow integrity by promoting local actin polymerization. J. Cell Biol. 182: 301–313. 10.1083/jcb.200712036 18644888 PMC2483530

[bib11] Carbone M. A. Yamamoto A. Huang W. Lyman R. A. Meadors T. B., 2016 Genetic architecture of natural variation in visual senescence in Drosophila. Proc. Natl. Acad. Sci. USA 113: E6620–E6629. 10.1073/pnas.1613833113 27791033 PMC5087026

[bib12] Cavieres M. F. Smith S. M., 2000 Genetic and developmental modulation of cardiac deficits in prenatal alcohol exposure. Alcohol. Clin. Exp. Res. 24: 102–109. 10.1111/j.1530-0277.2000.tb04559.x 10656199

[bib13] Chater-Diehl E. J. Laufer B. I. Singh S. M., 2017 Changes to histone modifications following prenatal alcohol exposure: An emerging picture. Alcohol 60: 41–52. 10.1016/j.alcohol.2017.01.005 28431792

[bib14] Debelak K. A. Smith S. M., 2000 Avian genetic background modulates the neural crest apoptosis induced by ethanol exposure. Alcohol. Clin. Exp. Res. 24: 307–314. 10.1111/j.1530-0277.2000.tb04612.x 10776667

[bib15] Dembeck L. M. Huang W. Carbone M. A. Mackay T. F. C., 2015 Genetic basis of natural variation in body pigmentation in *Drosophila melanogaster*. Fly (Austin) 9: 75–81. 10.1080/19336934.2015.1102807 26554300 PMC4826111

[bib71] Dembeck L. M. Huang W. Magwire M. M. Lawrence F. Lyman R. F., 2015 Genetic architecture of abdominal pigmentation in *Drosophila melanogaster*. PLoS Genet. 11: e1005163 10.1371/journal.pgen.1005163 25933381 PMC4416719

[bib16] Dietzl G. Chen D. Schnorrer F. Su K. C. Barinova Y., 2007 A genome-wide transgenic RNAi library for conditional gene inactivation in Drosophila. Nature 448: 151–156. 10.1038/nature05954 17625558

[bib17] Downing C. Flink S. Florez-McClure M. L. Johnson T. E. Tabakoff B., 2012 Gene expression changes in C57BL/6J and DBA/2J mice following prenatal alcohol exposure. Alcohol. Clin. Exp. Res. 36: 1519–1529. 10.1111/j.1530-0277.2012.01757.x 22530671 PMC3407322

[bib18] Edenberg H. J. Koller D. L. Xuei X. Wetherill L. McClintick J. N., 2010 Genome-wide association study of alcohol dependence implicates a region on chromosome 11. Alcohol. Clin. Exp. Res. 34: 840–852. 10.1111/j.1530-0277.2010.01156.x 20201924 PMC2884073

[bib19] Fish E. W. Murdaugh L. B. Sulik K. K. Williams K. P. Parnell S. E., 2017 Genetic vulnerabilities to prenatal alcohol exposure: Limb defects in sonic hedgehog and GLI2 heterozygous mice. Birth Defects Res. 109: 860–865. 10.1002/bdr2.1026 28504423 PMC5495621

[bib20] Fochler S. Morozova T. V. Davis M. R. Gearhart A. W. Huang W., 2017 Genetics of alcohol consumption in *Drosophila melanogaster*. Genes Brain Behav. 16: 675–685. 10.1111/gbb.12399 28627812 PMC5667673

[bib21] Gallant P., 2009 Drosophila Myc. Adv. Cancer Res. 103: 111–144. 10.1016/S0065-230X(09)03005-X 19854354

[bib22] Garic A. Berres M. E. Smith S. M., 2014 High-throughput transcriptome sequencing identifies candidate genetic modifiers of vulnerability to fetal alcohol spectrum disorders. Alcohol. Clin. Exp. Res. 38: 1874–1882. 10.1111/acer.12457 24962712 PMC4149215

[bib23] Garlapow M. E. Huang W. Yarboro M. T. Peterson K. R. Mackay T. F. C., 2015 Quantitative genetics of food intake in *Drosophila melanogaster*. PLoS One 10: e0138129 10.1371/journal.pone.0138129 26375667 PMC4574202

[bib24] Halder D. Mandal C. Lee B. H. Lee J. S. Choi M. R., 2015 PCDHB14- and GABRB1-like nervous system developmental genes are altered during early neuronal differentiation of NCCIT cells treated with ethanol. Hum. Exp. Toxicol. 34: 1017–1027. 10.1177/0960327114566827 25566775

[bib25] Hoyme H. E. May P. A. Kalberg W. O. Kodituwakku P. Gossage J. P., 2005 A practical clinical approach to diagnosis of fetal alcohol spectrum disorders: clarification of the 1996 Institute of Medicine criteria. Pediatrics 115: 39–47. 10.1542/peds.2004-0259 15629980 PMC1380311

[bib26] Hu Y. Flockhart I. Vinayagam A. Bergwitz C. Berger B., 2011 An integrative approach to ortholog prediction for disease-focused and other functional studies. BMC Bioinformatics 12: 357 10.1186/1471-2105-12-357 21880147 PMC3179972

[bib27] Huang D. W. Sherman B. T. Lempicki R. A., 2009 Systematic and integrative analysis of large gene lists using DAVID bioinformatics resources. Nat. Protoc. 4: 44–57. 10.1038/nprot.2008.211 19131956

[bib28] Huang W. Massouras A. Inoue Y. Peiffer J. Ramia M., 2014 Natural variation in genome architecture among 205 *Drosophila melanogaster* Genetic Reference Panel lines. Genome Res. 24: 1193–1208. 10.1101/gr.171546.113 24714809 PMC4079974

[bib29] Karak S. Jacobs J. S. Kittelmann M. Spalthoff C. Katana R., 2015 Diverse roles of axonemal dyneins in Drosophila auditory neuron function and mechanical amplification in hearing. Sci. Rep. 5: 17085 10.1038/srep17085 26608786 PMC4660584

[bib30] Kleiber M. L. Wright E. Singh S. M., 2011 Maternal voluntary drinking in C57BL/6J mice: advancing a model for fetal alcohol spectrum disorders. Behav. Brain Res. 223: 376–387. 10.1016/j.bbr.2011.05.005 21601595

[bib31] Kleiber M. L. Laufer B. I. Wright E. Diehl E. J. Singh S. M., 2012 Long-term alterations to the brain transcriptome in a maternal voluntary consumption model of fetal alcohol spectrum disorders. Brain Res. 1458: 18–33. 10.1016/j.brainres.2012.04.016 22560501

[bib32] Kojima T. Tsuji T. Saigo K., 2005 A concerted action of a paired-type homeobox gene, *aristaless*, and a homolog of Hox11/tlx homeobox gene, *clawless*, is essential for the distal tip development of the Drosophila leg. Dev. Biol. 279: 434–445. 10.1016/j.ydbio.2004.12.005 15733670

[bib33] Kopp A. Duncan I. Godt D. Carroll S. B., 2000 Genetic control and evolution of sexually dimorphic characters in Drosophila. Nature 408: 553–559. 10.1038/35046017 11117736

[bib34] Lacin H. Rusch J. Yeh R. T. Fujioka M. Wilson B. A., 2014 Genome-wide identification of Drosophila Hb9 targets reveals a pivotal role in directing the transcriptome within eight neuronal lineages, including activation of nitric oxide synthase and Fd59a/Fox-D. Dev. Biol. 388: 117–133. 10.1016/j.ydbio.2014.01.029 24512689 PMC4003567

[bib35] Lewohl J. M. Wang L. Miles M. F. Zhang L. Dodd P. R., 2000 Gene expression in human alcoholism: microarray analysis of frontal cortex. Alcohol. Clin. Exp. Res. 24: 1873–1882. 10.1111/j.1530-0277.2000.tb01993.x 11141048

[bib36] Liu J. Lewohl J. M. Harris R. A. Iyer V. R. Dodd P. R., 2006 Patterns of gene expression in the frontal cortex discriminate alcoholic from nonalcoholic individuals. Neuropsychopharmacol. 31: 1574–1582. 10.1038/sj.npp.1300947 16292326

[bib37] Logan-Garbisch T. Bortolazzo A. Luu P. Ford A. Do D., 2014 Developmental ethanol exposure leads to dysregulation of lipid metabolism and oxidative stress in Drosophila. G3 (Bethesda) 5: 49–59. 10.1534/g3.114.015040 25387828 PMC4291469

[bib38] Mackay T. F. C. Richards S. Stone E. A. Barbadilla A. Ayroles J. F., 2012 The *Drosophila melanogaster* Genetic Reference Panel. Nature 482: 173–178. 10.1038/nature10811 22318601 PMC3683990

[bib39] Mandal C. Park K. S. Jung K. H. Chai Y. G., 2015 Ethanol-related alterations in gene expression patterns in the developing murine hippocampus. Acta Biochim. Biophys. Sin. (Shanghai) 47: 581–587. 10.1093/abbs/gmv050 26063602

[bib40] Manning M. A. Eugene Hoyme H., 2007 Fetal alcohol spectrum disorders: a practical clinical approach to diagnosis. Neurosci. Biobehav. Rev. 31: 230–238. 10.1016/j.neubiorev.2006.06.016 16962173

[bib41] Maples T. Rothenfluh A., 2011 A simple way to measure ethanol sensitivity in flies. J. Vis. Exp. 48: 2541. 10.3791/2541 PMC3339835 21372791

[bib42] Marquardt K. Brigman J. L., 2016 The impact of prenatal alcohol exposure on social, cognitive and affective behavioral domains: Insights from rodent models. Alcohol 51: 1–15. 10.1016/j.alcohol.2015.12.002 26992695 PMC4799836

[bib43] McClure K. D. French R. L. Heberlein U., 2011 A Drosophila model for fetal alcohol syndrome disorders: role for the insulin pathway. Dis. Model. Mech. 4: 335–346. 10.1242/dmm.006411 21303840 PMC3097455

[bib44] McQuilton P. St Pierre S. E. Thurmond J. FlyBase Consortium, 2012 FlyBase 101–the basics of navigating FlyBase. Nucleic Acids Res. 40: D706–D714. 10.1093/nar/gkr1030 22127867 PMC3245098

[bib45] Memo L. Gnoato E. Caminiti S. Pichini S. Tarani L., 2013 Fetal alcohol spectrum disorders and fetal alcohol syndrome: the state of the art and new diagnostic tools. Early Hum. Dev. 89: S40–S43. 10.1016/S0378-3782(13)70013-6 23809349

[bib46] Mi H. Dong Q. Muruganujan A. Gaudet P. Lewis S., 2010 PANTHER version 7: improved phylogenetic trees, orthologs and collaboration with the Gene Ontology Consortium. Nucleic Acids Res. 38: D204–D210. 10.1093/nar/gkp1019 20015972 PMC2808919

[bib47] Minami R. Wakabayashi M. Sugimori S. Taniguchi K. Kokuryo A., 2012 The homeodomain protein defective proventriculus is essential for male accessory gland development to enhance fecundity in Drosophila. PLoS One 7: e32302 10.1371/journal.pone.0032302 22427829 PMC3299662

[bib48] Morozova T. V. Huang W. Pray V. A. Whitham T. Anholt R. R. H., 2015 Polymorphisms in early neurodevelopmental genes affect natural variation in alcohol sensitivity in adult Drosophila. BMC Genomics 16: 865 10.1186/s12864-015-2064-5 26503115 PMC4624176

[bib49] Nakagawa Y. Fujiwara-Fukuta S. Yorimitsu T. Tanaka S. Minami R., 2011 Spatial and temporal requirement of defective proventriculus activity during Drosophila midgut development. Mech. Dev. 128: 258–267. 10.1016/j.mod.2011.02.003 21376808

[bib50] Perkins A. Lehmann C. Lawrence R. C. Kelly S. J., 2013 Alcohol exposure during development: Impact on the epigenome. Int. J. Dev. Neurosci. 31: 391–397. 10.1016/j.ijdevneu.2013.03.010 23542005 PMC3703477

[bib51] Prigent C. Glover D. M. Giet R., 2005 Drosophila Nek2 protein kinase knockdown leads to centrosome maturation defects while overexpression causes centrosome fragmentation and cytokinesis failure. Exp. Cell Res. 303: 1–13. 15572022 10.1016/j.yexcr.2004.04.052

[bib52] R Core Team, 2016 R: A language and environment for statistical computing, R Foundation for Statistical Computing, Vienna.

[bib53] Quinn L. M. Secombe J. Hime G. R., 2013 Myc in stem cell behaviour: insights from Drosophila. Adv. Exp. Med. Biol. 786: 269–285. 10.1007/978-94-007-6621-1_15 23696362

[bib54] Richardson H. E. O’Keefe L. V. Reed S. I. Saint R., 1993 A Drosophila G1-specific cyclin E homolog exhibits different modes of expression during embryogenesis. Development 119: 673–690. 8187637 10.1242/dev.119.3.673

[bib55] Richardson H. O’Keefe L. V. Marty T. Saint R., 1995 Ectopic *cyclin E* expression induces premature entry into S phase and disrupts pattern formation in the Drosophila eye imaginal disc. Development 121: 3371–3379. 7588070 10.1242/dev.121.10.3371

[bib56] Riggs B. Rothwell W. Mische S. Hickson G. R. Matheson J., 2003 Actin cytoskeleton remodeling during early Drosophila furrow formation requires recycling endosomal components Nuclear-fallout and Rab11. J. Cell Biol. 163: 143–154. 10.1083/jcb.200305115 14530382 PMC2173427

[bib57] Rogic S. Wong A. Pavlidis P., 2016 Meta-analysis of gene expression patterns in animal models of prenatal alcohol exposure suggests role for protein synthesis inhibition and chromatin remodeling. Alcohol. Clin. Exp. Res. 40: 717–727. 10.1111/acer.13007 26996386 PMC5310543

[bib58] Roignant J. Y. Legent K. Janody F. Treisman J. E., 2010 The transcriptional co-factor Chip acts with LIM-homeodomain proteins to set the boundary of the eye field in Drosophila. Development 137: 273–281. 10.1242/dev.041244 20040493 PMC2799160

[bib59] Saito M. Chakraborty G. Hui M. Masiello K., 2016 Ethanol-induced neurodegeneration and glial activation in the developing brain. Brain Sci. 6:pii E31 10.3390/brainsci6030031 27537918 PMC5039460

[bib60] Sarmah S. Marrs J. A., 2017 Embryonic ethanol exposure affects early- and late-added cardiac precursors and produces long-lasting heart chamber defects in Zebrafish. Toxics 5: E35 10.3390/toxics5040035 29194345 PMC5750563

[bib61] Sauer K. Knoblich J. A. Richardson H. Lehner C. F., 1995 Distinct modes of cyclin E/cdc2c kinase regulation and S-phase control in mitotic and endoreduplication cycles of Drosophila embryogenesis. Genes Dev. 9: 1327–1339. 10.1101/gad.9.11.1327 7797073

[bib62] Schambra U. B. Goldsmith J. Nunley K. Liu Y. Harirforoosh S., 2015 Low and moderate prenatal ethanol exposures of mice during gastrulation or neurulation delays neurobehavioral development. Neurotoxicol. Teratol. 51: 1–11. 10.1016/j.ntt.2015.07.003 26171567 PMC4592804

[bib63] Schertel C. Huang D. Bjorklund M. Bischof J. Yin D., 2013 Systematic screening of a Drosophila ORF library in vivo uncovers Wnt/Wg pathway components. Dev. Cell 25: 207–219. 10.1016/j.devcel.2013.02.019 23583758

[bib64] Shcherbata H. R. Althauser C. Findley S. D. Ruohola-Baker H., 2004 The mitotic-to-endocycle switch in Drosophila follicle cells is executed by Notch-dependent regulation of G1/S, G2/M and M/G1 cell-cycle transitions. Development 131: 3169–3181. 10.1242/dev.01172 15175253

[bib65] Smith S. M. Garic A. Flentke G. R. Berres M. E., 2014 Neural crest development in fetal alcohol syndrome. Birth Defects Res. C Embryo Today 102: 210–220. 10.1002/bdrc.21078 25219761 PMC4827602

[bib66] Su B. Debelak K. A. Tessmer L. L. Cartwright M. M. Smith S. M., 2001 Genetic influences on craniofacial outcome in an avian model of prenatal alcohol exposure. Alcohol. Clin. Exp. Res. 25: 60–69. 10.1111/j.1530-0277.2001.tb02128.x 11198716

[bib67] Tulisiak C. T. Harris R. A. Ponomarev I., 2017 DNA modifications in models of alcohol use disorders. Alcohol 60: 19–30. 10.1016/j.alcohol.2016.11.004 27865607 PMC5420490

[bib68] Tunstall N. E. Herr A. de Bruyne M. Warr C. G., 2012 A screen for genes expressed in the olfactory organs of *Drosophila melanogaster* identifies genes involved in olfactory behaviour. PLoS One 7: e35641 10.1371/journal.pone.0035641 22530061 PMC3329464

[bib69] Tyler C. R. Allan A. M., 2014 Prenatal alcohol exposure alters expression of neurogenesis-related genes in an *ex vivo* cell culture model. Alcohol 48: 483–492. 10.1016/j.alcohol.2014.06.001 24954023 PMC4096774

[bib70] Yamamoto A. Zwarts L. Callaerts P. Norga K. Mackay T. F. C., 2008 Neurogenetic networks for startle-induced locomotion in *Drosophila melanogaster*. Proc. Natl. Acad. Sci. USA 105: 12393–12398. 10.1073/pnas.0804889105 18713854 PMC2527922

